# The search for attitude—a hidden curriculum assessment from a central European perspective

**DOI:** 10.1007/s00508-018-1312-5

**Published:** 2018-01-22

**Authors:** Birgit Ludwig, Bela Turk, Tamara Seitz, Isabella Klaus, Henriette Löffler-Stastka

**Affiliations:** 1 Dept. for Psychiatry and Psychotherapy, Medical University of Vienna, Vienna, Austria; 2 Kennedy Krieger Institute, Johns Hopkins Medical Institutions, Baltimore, USA; 3 Medical University of Vienna, Vienna, Austria; 4 Dept. for Psychiatry, SMZ Süd, Vienna, Austria; 5 Dept. for Psychoanalysis and Psychotherapy, and Teaching Center, Postgraduate Unit, Medical University Vienna, Währinger Gürtel 18–20, 1090 Vienna, Austria

**Keywords:** Occupational socialization, Medical students, Reflective capacity, Professionalism, Deprecation

## Abstract

**Background:**

Little is known about the development of the hidden curriculum in the medical education system. It refers to a conglomeration of implicit beliefs, attitudes and forms of conduct that are unwittingly transmitted from one generation of teaching physicians to the next. How can we describe this process, what are the potential positive or negative impacts, and last but not least, how can we measure it?

**Methods:**

Students of the Medical University of Vienna complete their clinical rotations in Vienna and in other accredited, mostly central European hospitals. They were subsequently invited to evaluate their rotations in an online questionnaire regarding dimensions, such as professionalism, teaching, integration and appreciation.

**Results:**

In total, 133 students participated in this pilot study and the average response rate was 10.1%, similar to evaluations conducted prior to that. Although the evaluation results on average were positive, several experiences of deprecation and less professional conduct were present in each evaluated rotation. Giving the students the opportunity to reflect upon their experiences could be seen as an intervention and investigation at the same time.

**Conclusions:**

This survey serves as a precursor to a qualitative interview-based study, accompanying the implementation of case-based learning designed by collaborating residents and medical students. The findings of this pilot-study support the necessity of fostering a reflective capacity in the education of medical students, enabling them to speak up and live up to the expected professionalism despite shortcomings within the hidden curriculum.

## Background

In the study course of the Medical Curriculum Vienna, the final years of studying medicine require clinical rotations throughout the departments of neurology, psychiatry, emergency medicine, pediatrics, gynecology and otorhinolaryngology/ophthalmology. Most of the students complete these rotations in teaching hospitals in the state of Vienna, a large portion of which are assigned to the Vienna General Hospital [1]. More recent curriculum changes have implemented accessible online platforms, engaging students through blended learning in an attempt to collectively approach and promote levels of knowledge, skill, and clinical reasoning [2–9]; however, medical education is more than a simple transmission of knowledge and skills; it is also a process of socialization [2]. Norms and values are transmitted to future physicians as they complete their final years of training. The hidden curriculum, taught (wittingly or unwittingly) in parallel to the defined curriculum goals, has been described as “processes, pressures and constraints which fall outside the formal curriculum, and which are often unarticulated or unexplored” [3].

This lack of articulation and verbalization within the student population as well as within the curriculum itself is mostly due to one of the two ways thoughts are formed. As Kahnemann claims in “Thinking, Fast and Slow”, the development of thoughts can be achieved through two different systems: through a fast and intuitive approach on the one hand and a slow and conscious process on the other. The second approach can be taken into account for many of our daily life choices, prone to heuristics and biases [4]. The mental pathways of slow rational thoughts is the one that ought to be embraced—it has to be nurtured and encouraged through institutionalized and structured teaching of reflective thinking [4, 5]. Reflective capacity and the development of mentalization is founded in (or at least tightly bound to) early attachment relationships, but stays modifiable throughout life; it is the predisposition for professional change (cf. mentalization-based therapy [10]). Prospective studies on attachment theory, going back over the past 30 years, show that the attachment style affects the ability to reflect. As defined by this theory, insecure-avoidant children would continue the relation to their attachment figure at the expense of their reflexive capacity [10]. The capacity to reflect not only influences patient-physician interactions [11], but also successful clinical reasoning [12]. Reflection in action [13] as Schön calls it, distinguishes the physician’s expertise to hold on for a minute and re-evaluate the thought process and decision-making; however, as already mentioned, any secondary socialization process that includes human interaction with an emphasis on the medical field, should foster and nurture a capacity to reflect. Interventions include reflective writing [14], such as text-based reflective journals or critical incident reports or creative use of digital media and storytelling [15]. The consensus of the medical education community is that the approach should be determined by the individual but should by all means be guided by a mentor person [16]. Short-term clinical reflection groups, such as Balint groups, would also be an asset supporting the professional development of medical students [16, 17].

The socialization process on the way to becoming a medical doctor constitutes implicit beliefs, transmitted attitudes, and observed behavior [18]. Those beliefs and attitudes stay implicit, transmitted over the fast pathway of thinking, as long as there is no structured, reflective approach of the curriculum. Mahood [2] even speaks of a hidden curriculum that one has to be “beware of”, although it does have both positive and negative sides to it.

Medical education research lacks data and assessment methods to describe this socialization process [19]. Although the medical curriculum learning objective contains attitude and professionalism, these variables are lacking precise operationalization. Consensus exists on the inclusion of teaching patient-centered care [1, 7–9] as a central aim, which at least is offered in the communication skills elements of the curriculum during the first 4 academic years. Current investigations [20] report a decrease of medical students’ intention to behave empathically in doctor-patient communication, patient-centered care or medical history taking during the course of study that cannot be ascribed to teaching content and cognitive training [21], but to contextual factors [3, 20] influencing professional development.

The aim of the exploratory observational study was to investigate the qualities of the hidden curriculum in a central European setting, assessing dimensions, such as professionalism in the context of a clinical rotation, and searching for hindering factors, such as degradation in a hierarchic system.

## Methods

In this pilot-study a total of 580 students were asked to complete an online questionnaire in order to evaluate their clinical rotations in the Vienna General Hospital (with a staff of over 9000 and over 2000 beds) and other affiliated teaching hospitals around the capital and other Austrian and European cities in the course of 2014.

The Vienna Hidden Curriculum (VHC) survey is in German (teaching language) and was approved by the Data Protection Committee and the Ethics Committee of the Medical University of Vienna. This data was collected anonymously online, using the online platform Moodle [22], which the students of the Medical University of Vienna have been familiar with since their very first year of studies. Students gave informed consent for participation.

The VHC survey assessed the clinical experiences of students in four dimensions. The dimensions, teaching and learning [2, 3], professionalism and communication [23, 24], hierarchy and integration [2, 3], social spheres and dignity/appreciation [2, 3, 23, 24] were assessed within 40 questions for each rotation. These dimensions and criteria are derivatives of significant findings in Lempp and Seale [3], Mahood [2], Sullivan et al. [24] and Kirk [23].

Students evaluated their rotations using a non-dichotomous ordinal scale ranging from minus 3 (disagree/very bad/not at all) to plus 3 (agree/very good/very often), allowing a graphic and more plastic grading of the completed rotations. Cut-off values were set on both ends of the questionnaire, both (−2), (−3) as well as (+2), (+3) were considered a decisive response; (−1), (0), (1) were considered indecisive.

Statistical analyses could be achieved through the open-source learning platform Moodle [22] allowing gathering of data and Microsoft Excel [25] in order to calculate average means.

## Results

A total of 133 students started responding to the survey; 51% of the students were in their last to final year and 49% were in their final year when responding to the questionnaires and their average age was 25.9 years. Of the respondents 47% were female and 53% were male (Table 1). The survey had different, department-specific parts and different response rates, listed in the following table. The average response rate was 10.1% (Table 2).

**Fig. 1:**
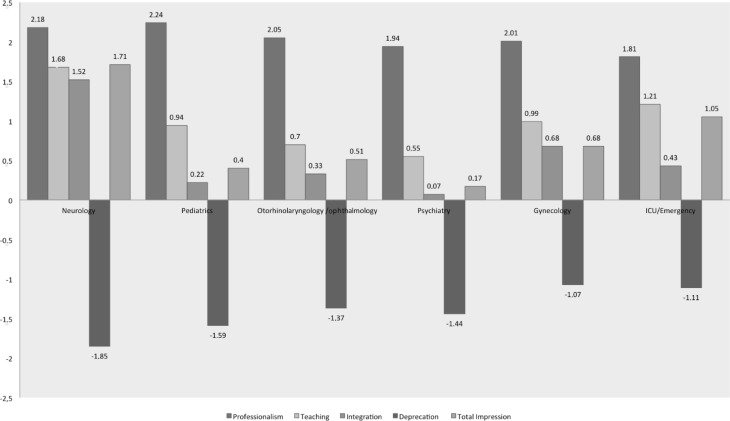
Results stratified by rotation, evaluated from −3 (disagree) to 3 (agree totally)

**Table 1:** Demographic numbers of the participants of the survey

Presumed graduation year	2015	2014	Total
	68	65	133
	0.51	0.49	100%
Sex	Female	Male	–
	62	71	133
	0.47%	0.53%	100%
Age (years)	25.9	–	–

**Table 2:** Responder rate depending on the rotation

	Neurology	Emergency medicine	Otorhinolaryngology/ophthalmology	Pediatrics	Psychiatry	Gynecology
Absolute	42	74	41	45	53	38
Percentage	7.2%	12.8%	7.1%	7.8%	9.1%	6.6%

Comparing the students’ impressions depending on the different rotations, one can see that the rating of this population of medical students concerning their learning experience, their impression of professionalism, their sense of integration, and their experiences of deprecation were homogeneously distributed (Table 3).

**Table 3:** Results stratified by rotation, evaluated from −3 (disagree) to +3 (agree totally)

	Professionalism (mean value)	Teaching (mean value)	Integration (mean value)	Deprecation (mean value)	Total Impression (mean value)
Neurology	2.18	1.68	1.52	−1.85	1.71
Pediatrics	2.24	0.94	0.22	−1.59	0.40
Otorhinolaryngology/ophthalmology	2.05	0.70	0.33	−1.37	0.51
Psychiatry	1.94	0.55	0.07	−1.44	0.17
Gynecology	2.01	0.99	0.68	−1.07	0.68
ICU/emergency	1.81	1.21	0.43	−1.11	1.05

The neurology rotation had the highest rating regarding the total impression (m = 1.71), teaching (m = 1.68), integration (m = 1.52). The students experienced less deprecation compared to other rotations (m = −1.85). Out of 42 responding students 4 did not feel appreciated or respected by the neurology staff.

Pediatrics had the highest rating concerning professionalism (m = 2.24), but lacked in successful integration of students in the hospital setting (m = 0.22). The total impression was on the neutral side (m = 0.40) and 7 out of 45 responding students (16%) did not feel appreciated or respected in their rotation.

Otorhinolaryngology/ophthalmology was rated rather highly on professionalism (m = 2.05) but poorly on teaching (m = 0.7). Despite the high level of professionalism, 2 students reported depreciative comments to patients in their presence and 4 students reported depreciative comments on absent patients. Out of 41 responding students 9 (22%) stated that they did not feel welcome at all.

The psychiatry rotation had the lowest rating concerning the total impression (m = 0.17), as well as teaching (m = 0.55) and integration (m = 0.07), reflecting rather neutral mean scores. Both the observed pleasure of teaching was rated very low by the students (m = 0.68), as well the general involvement of clinicians in the teaching process (m = 0.64), again reflecting a rather neutral mean score. Concerning the level of integration, 13 of the 53 responding students (25%) reported that they did not feel welcome at all and 8 out of 53 responding students (15%) did not feel appreciated at all.

The medical students of this population, by comparison, felt integrated (m = 0.68) in their gynecology rotation, but at the same time they experienced more deprecation than in other rotations (m = −1.07). Out of 38 responding students 7 (18%) did not feel appreciated at the gynecology department they were assigned to. One student reported racist comments in the absence and the presence of the patient and one other student reported the same for an absent patient.

The ICU rotation had the lowest rating on the professionalism level (m = 1.81), compared to the other rotations. Out of the 74 responding students 3 reported racist comments concerning patients, both in their absence and presence and 5 out of 74 responding students reported deprecating comments concerning the patients, both in their absence and presence. Additionally, 2 out of 74 responding students reported deprecating comments only in the absence of those patients, 7 students of the responders (9%) claimed they had witnessed deprecation of patients by the medical staff and 13 out of 74 (18%) responding students reported that they did not feel appreciated themselves during the rotation. Observing the mean score of the total impression, most students reported to have had a rather good rotation in the ICU/emergency departments (m = 1.05).

To give a résumé, 19.8% of the responding students did not feel appreciated or respected in their clinical rotation.

## Discussion

The average response rate of 10.1% is comparable to the response rates of evaluations conducted at the Medical University of Vienna. There was no significant difference in the response rate between the female and male students or students in their final year and students in their last to final year. The responders in this sample display a comparable distribution of age and gender as the population of medical students at the University of Vienna (Table 1); thus, this sample is representative for this population.

Although the evaluation results differed between the different rotations, experiences of deprecation and unprofessional conduct of teachers were present in all of them. For example, psychiatry having the lowest ratings corresponds to the well-established finding that students lose interest in and appreciation of this specialty after their rotation. Hypotheses include the emotional challenge as well as the annoyance of experiencing chronic disease [26, 27]. Citing how the students observed a lack of professionalism in their rotations is not a way of degrading the different rotations they have attended but a way of exposing a need for action as well as an intervention itself. In making the students think about the attitudes they observed and the professional conduct of their mentoring physicians, this survey helped them reflect upon their own attitude towards patients. What does it feel like to witness a physician making a racist joke in front of a patient with colored skin and would it feel differently for the students to do it themselves? If there is no delay, no moment of reflective thinking between observing a mentor’s attitude and acting, a “good” student would act the same. Phillips and Clarke conducted a qualitative interview study with Canadian medical students. They found the discrepancies between the politically correct constitutional curriculum and the implementation of it in training to be inspirational and reflection-triggering for these students [28].

Answering the questions of our survey made the students look at the discrepancies between their clinical rotation experiences and what professional conduct should be like according to the survey. The almost 20% of the students that claimed they did not feel respected during their rotation, realized that there was a discrepancy and were able to reflect upon it. Studies that were already cited show that their reflection process will allow them to have better patient-physician interactions [11] and also more successful clinical reasoning abilities. According to this statement, we suggest that there is need for a teaching setting that promotes professionalism, reflexive capacity, and insight skills. Training of insight and reflection skills is a popular topic in the field of psychotherapy research; findings show that the training of undergraduate students increased their self-efficacy for immediacy, challenges, and interpretation [29]. As educational sciences are based on neuroscientific findings [30] as well as on research on brain-based learning and teaching [31], and emotion theory, integrated thematic instructions [32] could be included to respect the affect-cognitive interface in teaching and learning processes. In his publication on therapists’ competencies, Caspar referred to aspects of conscious analytical processing within intuitive processing, gained by factor analysis. Variables that loaded high on the conscious analytical processing factor include application of rules, conscious processing, reflecting reasons for procedures, meta-analytic information processing and search for alternatives [12]. These results could be applied to graduate medical student training as well, such as in guideline confirmative behavior. Examples in bedside teaching include taking a moment of reflection before deciding which imaging procedure one needs instead of taking all three available ones or taking a step back in the construction of a differential diagnosis [6, 7].

Other potential interventions include external or internal teaching evaluations [33–35] as well as additional support specifically for teaching clinicians as described by Foster and Laurent [36].

Since the teaching system differs from country to country it might be important to shed light on the specifics that shape the Austrian model of medical education [21]. Shortcomings in the postgraduate education might be a reflection of the ability of junior doctors to teach medical students during their rotations [37, 38]. If the presented problem is at least partially due to this top-down process, interventions in the postgraduate education system might be required to improve the quality of bedside teaching in medical school.

Another point that should be discussed is the result of the question “did you feel appreciated/respected on the floor of your clinical rotation?” Almost 20% of the responding students stated that they did not feel appreciated or respected at all. Can we transfer this finding to the whole population of our medical students? Is there a bias in the responder rate? One could argue that students who had bad experiences were more prone to answer this survey than those who appreciated their rotations.

The results of this pilot study are directing us towards a qualitative interview approach, allowing us to elucidate the mechanisms of the hidden curriculum as well as successful learning of clinical reasoning in a bedside setting. In practical terms for example, case-based eLearning courses [9] could be used for the preparation of structured case presentations, teachers and students could then be consistently prepared for bedside teaching [8] and their qualitative interview-based evaluation [39, 40]. On the general level, thematic analyses, narrative synthesis of quantitative mediator outcome studies [41] or synthetic reviews could be undertaken to foster critical thinking skills and provide teachers and students with frameworks to understand the broader historical, structural, and cultural patterns that influence healthcare interactions [42] and the impact of role modelling, mentorship [43] and learning [42, 44].

## Conclusion

Learning within the setting of a clinical rotation will bring challenging factors, such as non-confirmative behavior of the instructors or lack of professionalism of the instructors. Providing a structured and institutional approach of reflection would enable the students to overcome these hindrances. Our findings showing discrepancies between the constituted curriculum and its implementation in training, support the necessity of fostering reflective capacity in the education of medical students and show that there is a need for a positive reinforcement in order to facilitate the learning process and assure successful clinical reasoning in these future physicians. An effective diagnostic or therapeutic interaction between patient and doctors consists of more than words. Emotional communication and empathic understanding influence patient satisfaction and affect outcome. To train these ingredients, a proper learning environment providing containment and mentalization is necessary.

### Lessons for the practice

- Fostering reflective capacity in the education of medical students promotes cognitive learning processes.
- Deprecation in the context of a clinical rotation is a hindering factor for cognitive learning.
- Interventions include Balint groups, mentor-guided reflection tasks, attitude training and verbalized meta-analytic processing of mentors.
- Giving the students the opportunity to reflect upon their experiences can be seen as an intervention and investigation at the same time.
